# Temperature-related growth limits and wood decay capacity of the warmth-loving fungus *Biscogniauxia nummularia in vitro*

**DOI:** 10.3389/ffunb.2025.1548128

**Published:** 2025-04-11

**Authors:** Jan Tropf, Steffen Bien, Johanna Bußkamp, Holger Sennhenn-Reulen, Johanna Becker, Jörg Grüner, Gitta Jutta Langer, Ewald Johannes Langer

**Affiliations:** ^1^ Forestry Research and Competence Centre (FFK Gotha), ThüringenForst, Gotha, Germany; ^2^ Department of Forest Protection, Northwest German Forest Research Institute (NW‐FVA), Göttingen, Germany; ^3^ Faculty of Mathematics and Natural Sciences, Institute for Biology, Department of Ecology, University of Kassel, Kassel, Germany; ^4^ Department of Forest Growth, Northwest German Forest Research Institute (NW‐FVA), Göttingen, Germany; ^5^ Department of Forest Protection, Forest Research Institute of Baden Württemberg (FVA BW), Freiburg, Germany

**Keywords:** *Biscogniauxia nummularia*, temperature-related growth, wood decay, Germany, *Fagus sylvatica*, Vitality Loss of Beech

## Abstract

Temperature-related growth characteristics and wood decay capacities of *Biscogniauxia nummularia* strains were analysed *in vitro*, revealing variability between strains. To model the growth characteristics fitted dose-response curves were generated using the four-parameter Brain-Cousens hormesis model. The different strains showed distinct optimum growth temperatures, with some achieving maximum growth at 25°C, while others peaked at 28°C, depending on the tested culture media. Strains tested also exhibited variation in their temperature ranges for measurable growth, with some tolerating a broader range than others. The results of the study lead to the consideration that temperature tolerance as well as the optimal growth temperature might be influenced by the strains’ geographic origin, with those from southern Germany possibly adapted to higher temperatures. In terms of wood decay, mass loss caused by the various strains differed clearly in many cases, suggesting potential strain-dependent differences in pathogenicity. Additionally, genetic analysis of the beta-tubulin DNA region of *B. nummularia* specimens examined revealed considerable variations between the strains.

## 1 Introduction

*Biscogniauxia nummularia* (Bull.) Kuntze (*Graphostromataceae*, *Xylariales*, *Ascomycota*, Wendt et al., 2018), is considered one of the most abundant endophytes of European beech (*Fagus sylvatica* L.) (Chapela and Boddy, 1988; Langer and Bußkamp, 2021; Langer et al., 2021; Tropf et al., in press). Although *B. nummularia* has been detected as an endophyte on various coniferous and deciduous trees (*e.g.* Bußkamp, 2018; Peters et al., 2023; Schlößer et al., 2023), according to the current knowledge, it only fructifies and occurs as a pathogen on European beech and Oriental beech (*Fagus orientalis* Lipsky) (Petrini and Petrini, 1985; Nugent et al., 2005; Zamani et al., 2024). When the host (*Fagus*) comes under environmental stress, *e.g.* due to drought and heat, *B. nummularia* can switch from its endophytic lifestyle into a pathogenic phase and cause various symptoms ranging from bark necroses and strip-cankering to wood decay and beech decline (Granata and Whalley, 1994; Hendry et al., 1998, 2002; Granata and Sidoti, 2004; Nugent et al., 2005; Luchi et al., 2015).

With the exception of 2021, the years between 2018 and 2022 were exceptionally dry and hot in Germany (Rakovec et al., 2022; Imbery et al., 2023), which led to the widespread occurrence of Vitality Loss of Beech (VLB) (Langer, 2019; John et al., 2019; Langer et al., 2020; Langer and Bußkamp, 2023). This complex disease of European beech is primarily caused by abiotic factors, but the damage progression is strongly influenced by accompanying fungi and insects (Bressem, 2008; Lakatos and Molnár, 2009; Brück-Dyckhoff et al., 2019; Langer and Bußkamp, 2021, 2023). Studies have shown that the current VLB outbreak, which started in 2018, is strongly associated with anamorphic and teleomorphic fructifications of *B. nummularia* (Langer and Bußkamp, 2021, 2023). Hendry et al. (1998) reported that *B. nummularia* continues to grow rapidly at 30°C. It is therefore not surprising that this fungus, which is sensitive to reductions in host vitality, is involved in a complex disease that is primarily triggered by high temperatures and drought. The average number of days (d) with daytime maximum of at least 30°C (hot days) was 20.4 d in 2018 and 17.0 d in 2019. For comparison, this value was 4.2 d for the reference period 1961 to 1990 (https://opendata.dwd.de/climate_environment/CDC/grids_germany/multi_annual/air_temperature_mean/DESCRIPTION_gridsgermany_multi_annual_air_temperature_mean_6190_en.pdf).

Reports of European beech decline associated with *B*. *nummularia* and drought are not confined to Germany. There are reports of high mortality and beech decline associated with *B*. *nummularia* from Hungary (Lakatos and Molnár, 2009), Italy (Granata and Sidoti, 2004), and France (Mirabel and Gaertner, 2023). Vujanovic et al. (2020) declared identification of a hybrid of *Biscogniauxia anceps* (Sacc.) J.D. Rogers, Y.M. Ju & Cand. and *B. nummularia* isolated in Montenegro, and proposed a new species based on an analysis of morphological and molecular data. The name *Biscogniauxia destructiva* Vujan was introduced due to its aggressiveness towards European beech, however, due to violation of the International Code of Nomenclature for algae, fungi, and plants the name is listed as invalid (Art. F.5.1 Shenzhen^1^). Further studies from Europe, for example from England and Wales (Nugent et al., 2005), Spain (Zabalgogeazcoa et al., 2015), the Czech Republic (Zíbarová and Kout, 2017), and Poland (Patejuk et al., 2022), confirm the widespread presence of *B*. *nummularia* in European beech. While the ability of Ascomycota to cause mass loss through wood decay is typically considered limited in comparison to Basidiomycota, it is well documented that species of the *Xylariales* are capable of causing severe mass loss (Merrill et al., 1964; Duncan and Eslyn, 1966; Worrall et al., 1997). Using microscopic cross-sections of heavily infected branches, Weber and Mattheck (2009) were able to verify that *B*. *nummularia* causes soft rot. The cells of the affected areas revealed cavities in the secondary cell walls, which speaks in favour of a type-I soft rot (Savory, 1954; Corbett, 1965). The convergence of these cavities led to an increasing degradation of the cellulose-rich S2 layer of the cell wall. In the final stage, only the primary wall and the middle lamella remained. As a result of the decay, branches with leaves still attached to them, are reported to break off. The fractures were brittle (Weber and Mattheck, 2009; Langer et al., 2020).

Fungal growth and the associated depolymerisation of lignocellulose in wood are primarily influenced by wood moisture and temperature, along with the interaction between both factors (Viitanen and Ritschkoff, 1991; Viitanen, 1997; Brischke and Rapp, 2008; Goodell, 2020). However, the optimum growth temperature for *B. nummularia* has never been determined in the past. Studies by Tropf et al. (2022) indicate that the wood decay capacities of various *B. nummularia* strains differ considerably *in vitro*. It is known that even under controlled laboratory conditions, different fungal strains of the same species can differ in growth rate, optimum growth temperature, and secondary metabolism production (Schwarze, 1992; Sørensen and Giese, 2013; Dresch et al., 2015). This led to the consideration that different strains of *B. nummularia* may have different optimum temperatures with regard to hyphal growth. The present study was conducted to verify the initial results of Tropf et al. (2022) concerning the wood decay capacities of various strains of *B*. *nummularia* from different parts of Germany. Temperature- and nutrient medium-related growth characteristics of the selected *Biscogniauxia* strains were examined *in vitro*. The optimum growth temperature and both the highest and lowest temperature at which the respective strain still showed measurable growth was determined (cardinal temperature). Furthermore, the temperature above the optimum temperature for each strain that resulted in the demise of the culture was identified.

The following hypotheses were formulated: 1) *B*. *nummularia* strains can differ in their temperature-related growth characteristics, 2) Wood decay capacities of *B*. *nummularia* strains can vary considerably, and 3) *B*. *nummularia* strains originating from host trees other than European beech cause considerably less mass loss on European beech wood compared to strains originating from European beech.

## 2 Materials and methods

### 2.1 Selection of fungal strains

**Figure 1:**
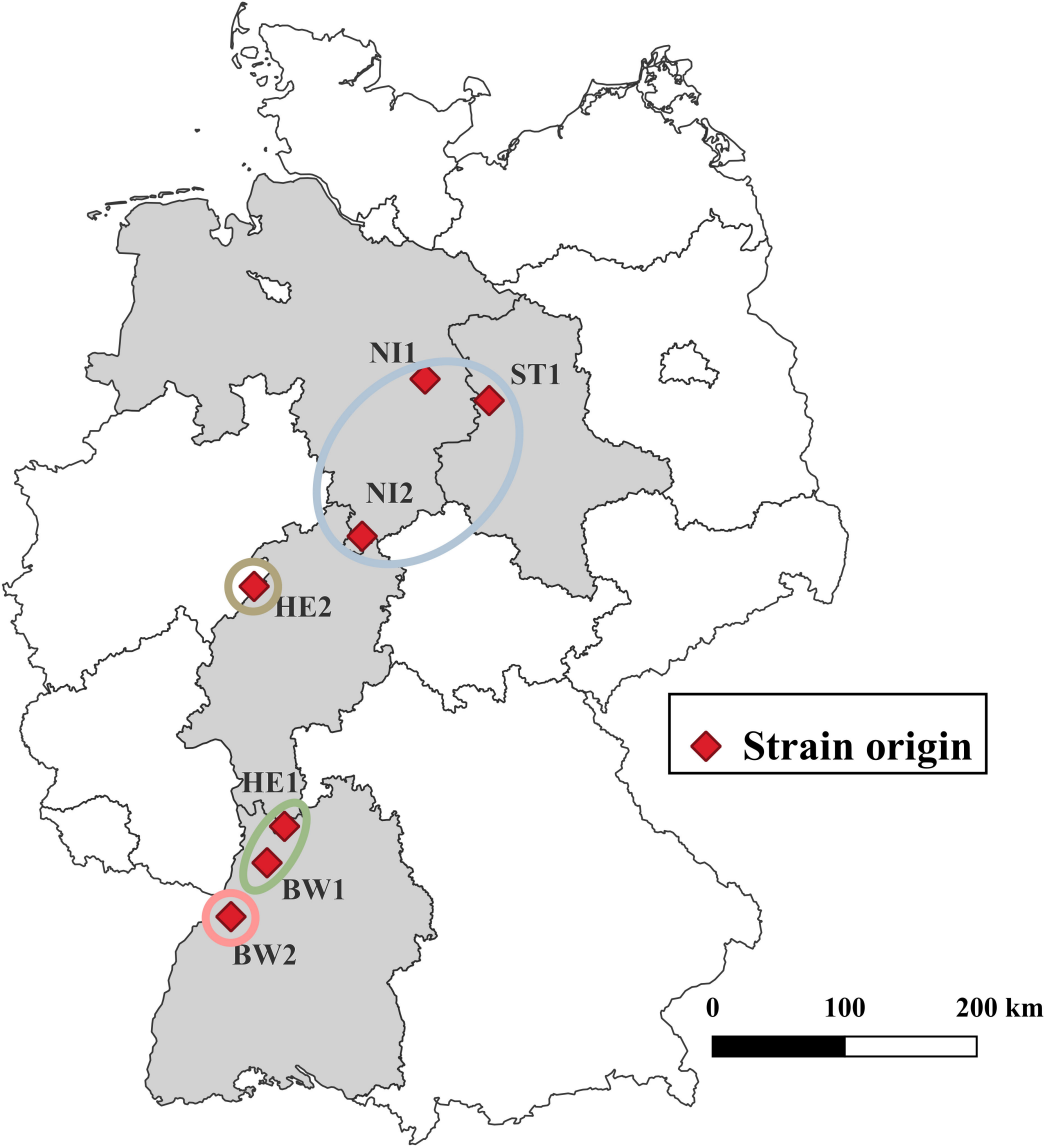
Origin of the various *Biscogniauxia nummularia* strains. The strains are grouped according to the illustration in **Supplementary Figure 1**. ^©^ GeoBasis-DE/BKG 2023 for boundaries of Germany and the federal states.

All seven *B*. *nummularia* strains used were derived from the fungal culture collection of the Northwest German Forest Research Institute growing on Malt Yeast Peptone Agar (MYP), modified according to Langer (1994) (0.7% malt extract (Merck, Darmstadt, Germany), 0.05% yeast extract (Merck), 0.1% peptone (Merck) and 1.5% agar (Merck)). The strains originated from European beech (NI2, HE2, BW1, BW2), Douglas fir (*Pseudotsuga menziesii* (Mirbel) Franco, HE1), and Scots pine (*Pinus sylvestris* L., NI1, ST1) across seven forest stands in Germany (**Table 1**; **Figure 1**). Strains derived from Douglas fir and Scots pine were included to test whether they differ from beech strains in their ability to cause mass loss in European beech wood. All strains were isolated either from tree compartments or ascocarps. Isolates were obtained during causal analyses of damaged forest trees in Germany, prior to this study. So all host trees exhibited declines in vitality during the sampling period. For the years 1991–2020, data was obtained from the nearest weather stations of every locality where the strains were isolated^2^ (**Supplementary Table 1**).

**Table 1:** Data of the *Biscogniauxia nummularia* strains.

Strain	ID NW-FVA	Year of isolation	Host	Origin	Federal state	WGS 84	NCBI-Accession No. ITS	NCBI-Accession No. *TUB*
ST1	4756	2018	*Pinus sylvestris*	Branch	Saxony-Anhalt	N: 52.3268 E: 11.1889	PQ722107	PQ757296
NI1	6186	2021	*Pinus sylvestris*	Branch	Lower Saxony	N: 52.4847 E: 10.4813	PQ722108	PQ757297
NI2	8204	2022	*Fagus sylvatica*	Fruiting body	Lower Saxony	N: 51.4199 E: 9.7605	PQ722109	PQ757298
HE1	8234	2022	*Pseudotsuga menziesii*	Branch	Hesse	N: 49.4451 E: 8.9174	PQ722110	PQ757299
HE2	8951	2022	*Fagus sylvatica*	Trunk	Hesse	N: 51.0800 E: 8.5861	PQ722111	PQ757300
BW1	9899	2019	*Fagus sylvatica*	Branch	Baden-Württemberg	N: 49.1964 E: 8.7363	PQ722112	PQ757301
BW2	9900	2020	*Fagus sylvatica*	Branch	Baden-Württemberg	N: 48.8276 E: 8.3659	PQ722113	PQ757302

### 2.2 Molecular analysis

Previous to molecular analysis, the strains were morphotyped according to Bußkamp et al. (2020). For species identification DNA extraction as well as amplification of the 5.8S nuclear ribosomal gene with the two flanking internal transcribed spacers ITS-1 and ITS-2 (ITS region) was carried out for all strains following Tropf et al. (acc.). In addition, various primer combinations were used to obtain as complete a picture as possible of the beta-tubulin gene region (*TUB*) of the strains used. Primers Bt1a, Bt1b, Bt2a, Bt2b (Glass and Donaldson, 1995), T1, T2, and T22 (O’Donnell and Cigelnik, 1997) were employed applying PCR conditions of (Paulin and Harrington, 2000). A StepOnePlus™ PCR System (Applied Biosystems, Waltham, Massachusetts, US) or a GeneExplorer 96 (Hangzhou BIOER Technology, Hangzhou, China) was used to carry out the DNA amplifications. After visualisation in 1% agarose gel, PCR products were sent to Eurofins Scientific Laboratory (Ebersberg, Germany) for sequencing. From all resulting sequences consensus sequences were generated, and visually checked and edited if necessary using BioEdit Sequence Alignment Editor (v. 7.2.5; (Hall, 1999)). Sequence regions generated by different *TUB* primer combinations were joined at the overlap points, using Geneious R11 (Kearse et al., 2012). Sequences were submitted to GenBank (**Table 1**). An ITS sequence dataset was compiled from sequences generated in this study and sequences analysed by Vujanovic et al. (2020). The ITS sequence dataset and a *TUB* dataset consisting of *TUB* sequences generated in this study were aligned automatically using MAFFT v. 1.5.0 (Katoh, 2002; Katoh and Standley, 2013) implemented in Geneious R11 and manually adjusted where necessary. Maximum Likelihood analyses were performed by RAxML v. 4.0 (Stamatakis, 2006, 2014) using the GTRGAMMA model with the rapid bootstrapping and search for best scoring ML tree algorithm including 1000 bootstrap replicates implemented in Geneious R11, respectively.

### 2.3 Determination of the temperature-related growth and mortality

The growth characteristics of the seven investigated *B. nummularia* strains were tested on four different culture media at temperature gradients from 0°C to 7°C and 25°C to 36°C at 1 degree intervals. For this purpose, Petri dishes (90 mm diameter) with the following media were inoculated with one of the strains each to be tested on the centre of each dish: MYP, Malt Extract Agar (MEA, 2% malt extract (Merck) and 1.6% agar (Merck)), Potato Dextrose Agar (PDA, 0.4% Potato extract (Sigma Aldrich, Steinheim, Germany), 2% Dextrose (Sigma Aldrich) and 1.5% Agar (Sigma Aldrich)) or Synthetic Nutrient-Poor Agar (SNA, 0.1% KH_2_PO_4_ (Merck), 0.1% KNO_3_ (Sigma Aldrich), 0.05% MgSO_4_*H_2_O (Sigma Aldrich), 0.05% KCl (Merck), 0.02% Glucose (Sigma Aldrich), 0.02% Sucrose (Sigma Aldrich) and 2% Agar (Merck)). The inoculated Petri dishes were placed in climate chambers (KBW E6, Binder, Tuttlingen, Germany). To determine the growth optimum and the temperature at which the strains showed no measurable growth, the temperature gradient from 25°C to 36°C was tested. Temperatures between 25°C and 36°C were selected based on the findings of Hendry et al. (2002), who demonstrated that *B*. *nummularia* can still exhibit rapid growth at 30°C. Consequently, this temperature was used as the initial reference point in the experiment, with additional temperatures tested above and below this value. For each strain, two inoculated Petri dishes were tested per temperature and culture medium. Originally, an incubation period of one week was planned to test this temperature range. However, depending on the medium and temperature, the surface of the medium in the Petri dishes was already completely covered after one week. So after four days of incubation, the inoculated Petri dishes were already removed from the climate chambers and the extent of the hyphal growth was measured in mm with a ruler. The methodology for determining growth based on the diameter of the fungal colony was derived from the work of Brancato and Golding (1953). Two orthogonal straight lines were drawn on the undersides of the Petri dishes, which intersected at the inoculation point. Hyphal growth was measured along the four axes created and the values averaged (arithmetic mean). To determine the temperature, above the maximum growth temperature, at which the fungus dies, one petri dish per strain and culture medium was prepared in triple repetition, and incubated at temperatures 36°C and 37°C, respectively. After one week, two weeks, and three weeks each one culture for every medium was transferred to room temperature (approx. 22°C), without direct sunlight. If no growth of the fungal strain was observed after an additional 14 days incubation under these conditions, the culture was assumed to be dead.To assess the lowest temperature at which the *B. nummularia* strains still showed measurable growth, one inoculated Petri dish per strain, culture medium and temperature was incubated in climate chambers at temperatures between 0°C and 7°C for 14 days. The experiment was started at 0°C, and continued with increasing temperatures until all of the tested strains exhibited visible growth.

### 2.4 Determination of the wood decay capacities

The wood decay capacities of the *B. nummularia* strains tested were investigated in a test based on the DIN standard on the durability of wood and wood-based products – Wood preservatives – Method of test for determining the protective effectiveness against wood destroying basidiomycetes – Determination of the toxic values, German version (DIN EN 113, 1996). For the production of the wooden test objects (TOs), sapwood of the trunk from a freshly harvested beech was cut into cuboids measuring 5 cm · 2.5 cm · 1.5 cm (length · width · height). TOs with obvious wood defects were sorted out and excluded from the test. To determine the initial dry mass, the TOs were dried to constant weight in a drying oven (UM 500, Memmert, Schwabach, Germany), at 103°C for 24 hours. The weight of the TOs was determined to an accuracy of 0.001 g and defined as the initial dry mass *m_0_* before incubation. The TOs were then soaked in tap water for 24 hours and afterwards sterilised in an autoclave at 121°C for 20 minutes (VARIOKLAV^®^ 400EP, HP Labortechnik GmbH, Oberschleißheim, Germany). Duran^®^ square bottles (SCHOTT AG, Mainz, Germany) with membrane caps (test vessels) were filled with 80 ml MEA, autoclaved at 121°C for 20 minutes and then stored horizontally. Mycelium of strains tested, pre-cultivated on MEA, was transferred to the test vessels using an inoculation loop. Two inoculation points were chosen, one at the front of the bottle and one at the back. Control vessels also contained 80 ml MEA but were not inoculated. After the surface of the culture medium in the vessels was completely covered with a fungal strain, three sterile TOs were added to each test vessel, including control vessels. The incubation periods were set at six and nine weeks. For each incubation period, five test vessels were prepared for each strain plus five test vessels for control (corresponds to 15 TOs per treatment group). All test vessels were stored in the dark in a climate-controlled room where the temperature was set to 25°C. The air temperature was measured constantly over the incubation periods using a HOBO data logger (Onset, Bourne, USA). After each incubation period, the TOs were taken out of the test vessels and the mycelium on the surface of the TOs was removed with a razor blade. Thirteen TOs per treatment group were used to determine the wood decay capacities. The remaining two TOs per treatment group were excluded from the present study and used for the preparation of histological sections (not shown). The TOs were then dried at 103°C for 24 hours. The weight of the TOs was determined again to an accuracy of 0.001 g and defined as the final dry mass *m_3_* after incubation. The wood decay capacities was equated with relative mass loss of the TOs after the incubation period. The formula for calculating the relative mass loss was based on DIN EN 113 (1996):


$$RML=\;\frac{m_{0}-m_{3}}{m_{0}}\;\cdot 100\;\left[\%\right]$$

*RML*: Relative mass loss of the TO after the incubation period [%].

$m_{0}$ : Initial dry mass of the TO before incubation [g].

$m_{3}$ : Final dry mass of the TO after incubation [g].

### 2.5 Data analyses

**Figure 2:**
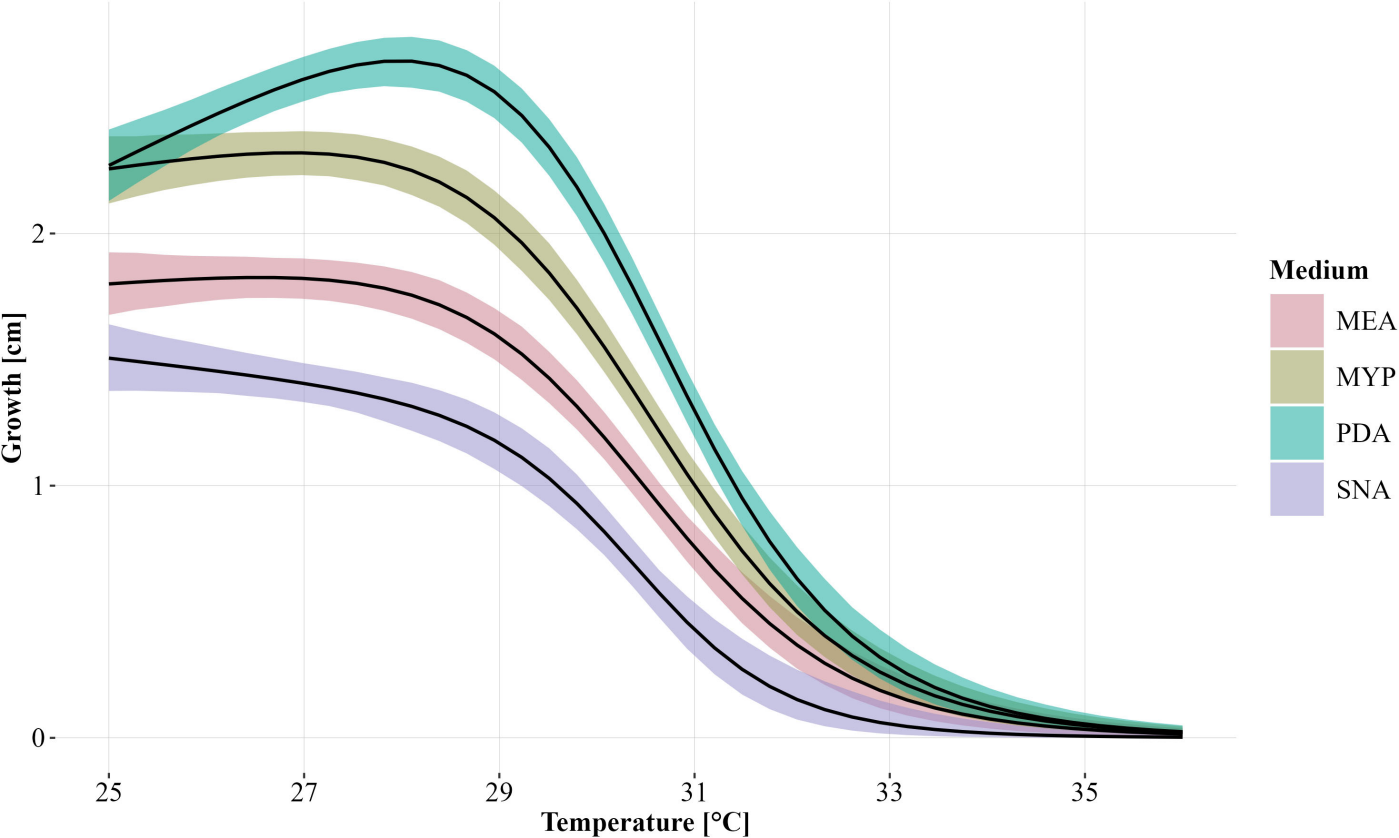
Modelled growth of *Biscogniauxia nummularia* as a function of temperature and grouped according to the culture media (MEA, Malt Extract Agar; MYP, Malt Yeast Peptone Agar; PDA, Potato Dextrose Agar; SNA, Synthetic Nutrient-Poor Agar) used without differentiation of the strains. The fitted dose-response curves were generated using the four-parameter Brain-Cousens hormesis model. The 95% credible interval is shown for each group. Growth was measured after four days of incubation.

Statistical differences in relative mass loss caused by the different strains were investigated using beta regression models and Tukey multiple comparisons. We do not convert statistical inference statements based on p-values into binary test decisions, but rather use p-values as a continuous measure of evidence (Wasserstein, 2019). Where possible, the associated effect sizes are reported and evaluated in the applied context. To model the radial growth of the different strains at temperatures from 25°C to 36°C, dose-response curve models were fitted using the four-parameter Brain-Cousens hormesis model (95% credible intervals shown in **Figure 2**). Data was analysed using the statistical software environment R (R Core Team, 2024, version 4.4.1), with using R add-on packages betareg (Cribari-Neto and Zeileis, 2010), dplyr (Wickham et al., 2023), drc (Ritz et al., 2015), emmeans (Lenth et al., 2024), ggplot2 (Wickham, 2016), MASS (Venables and Ripley, 2002), and plyr (Wickham, 2011).

## 3 Results

### 3.1 Results of temperature-related growth and mortality

Temperature and the used culture medium had a strong influence on the growth of the *B. nummularia* strains. Comparing the culture medium, the lowest growth across all fungal strains between 25°C and 34°C, was observed for SNA (**Figure 2**). Strain HE2 did already show no measurable growth at 32°C within the four days of incubation on SNA. For the other six strains, the maximum temperature at which the strain still showed growth on SNA varied between 32°C and 33°C (**Table 2**). On the culture media other than SNA, there were strains that still grew out at 34°C and strain NI2 still showed growth on PDA even at 35°C. However, at 34°C, growth on all strains and independent of the culture media was at most very low. At 36°C, no strain showed growth within the incubation period regardless of the culture medium used. At 25°C, the measured growth across all fungal strains was almost equal between PDA and MYP, but with increasing temperatures up to 30°C, the growth measured for the fungal strains on PDA got increasingly higher in comparison to MYP. At 31°C, the average growth on the two culture media began to converge, and the difference between them decreased as the temperature increased further. For the MEA culture medium the growth values across all fungal strains were always located between MYP and SNA at temperatures between 25°C and 33°C.

**Table 2:** Temperature-related growth characteristics of *Biscogniauxia nummularia* strains.

Strain	MEA	MYP	PDA	SNA
Temperature with maximal growth [°C]				
ST1	25	25	25-27	27
NI1	25-26	27	28	27-28
NI2	28	27-28	27	25; 28-29
HE1	27	25	27	27
HE2	25	25	27	25
BW1	28	27	28	26
BW2	28	28	28	25-26
Highest temperature with measurable growth [°C]				
ST1	34	33	34	32
NI1	31	32	32	33
NI2	34	34	35	33
HE1	33	34	34	32
HE2	33	33	34	31
BW1	34	34	34	33
BW2	33	32	33	32
Lowest temperature with measurable growth [°C]				
ST1	3	3	4	3
NI1	6	6	6	6
NI2	4	4	3	4
HE1	5	5	5	5
HE2	3	3	4	3
BW1	5	5	5	5
BW2	6	6	6	6

Growth characteristics of the strains BW1-2, HE1-2, NI1-2 and ST1 were determined in culture on different artificial media (MEA, Malt Extract Agar; MYP, Malt Yeast Peptone Agar; PDA, Potato Dextrose Agar; SNA, Synthetic Nutrient-Poor Agar). Maximal growth and highest temperature with measurable growth was determined after four days, lowest temperature with measurable growth was determined after two weeks.

**Figure 3:**
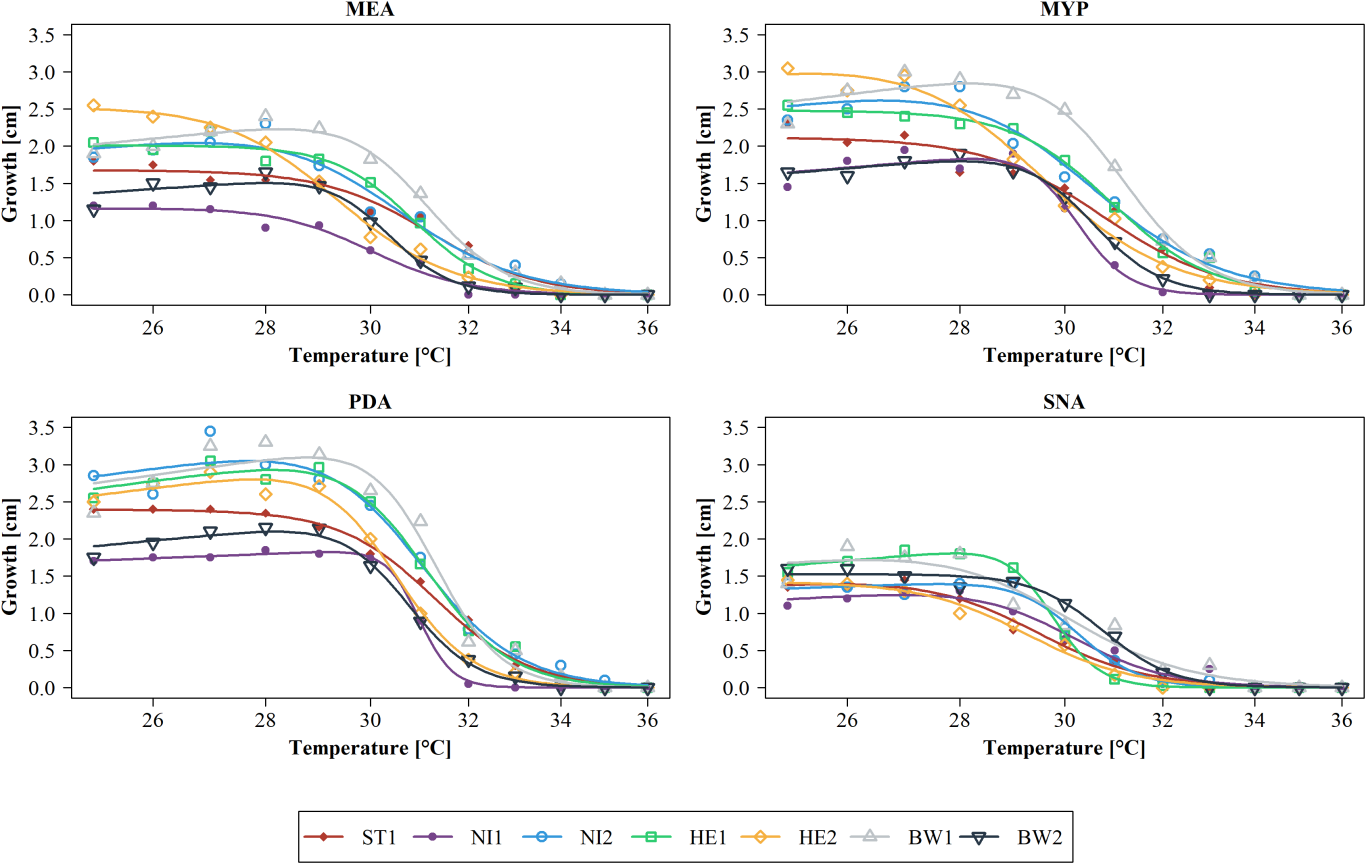
Modelled growth per *Biscogniauxia nummularia* strain (BW1-2, HE1-2, NI1-2 and ST1) as a function of temperature and subdivided according to the culture media used (MEA, Malt Extract Agar; MYP, Malt Yeast Peptone Agar; PDA, Potato Dextrose Agar; SNA, Synthetic Nutrient-Poor Agar). Dose-response curves were fitted using the four-parameter Brain-Cousens hormesis model. Actual measured growth values (n = 2 per strain, temperature, and culture medium) were averaged and are presented as dots. Growth was measured after four days of incubation.

If the modelled temperature-related growth depending on the media is considered individually for each strain, it is noticeable that the growth characteristics differ clearly (**Figure 3**). Strain ST1 already reached its maximum growth at 25°C on MEA, MYP and PDA (**Table 2**), but the growth curve declined slowly at rising temperatures compared to other strains like strain HE2. On SNA, the growth of strain ST1 peaked at 27°C. Strain NI1 showed low growth on all culture media compared to the other strains. On MEA, growth of this strain culminated at 25°C and 26°C, after which growth declined rather evenly. On MYP, PDA and SNA, growth culminated at 27°C and 28°C, but growth dropped rapidly for MYP and PDA in particular at 30°C and 31°C. The growth of strain NI2 peaked rather late on all culture media (27°C or 28°C) except SNA and showed comparatively high growth even at temperatures above 30°C, until the growth curves converge at 33°C at the latest for all strains. At a temperature of 27°C on PDA, the two cultures of strain NI2 achieved the highest measured growth in the study at 3.6 cm and 3.7 cm. Strain HE1 showed similar growth characteristics to strain NI2 on MEA, MYP and PDA, but the measured growth between 26°C and 28°C was usually slightly lower than that of strain NI2. On MYP, the growth of strain HE1 already culminated at 25°C, and then declined slowly. On SNA, the measured growth was comparatively high up to 29°C and then dropped rapidly. Strain HE2 reached its maximum growth on MEA and MYP at 25°C. At this temperature, the measured growth was also considerably higher compared to the other strains. Above 25°C, the measured values for HE2 decreased more or less steadily. From 30°C onwards, the strain showed only low growth on the two culture media compared to many other strains. On PDA, HE2 culminated at 27°C and growth at 25°C was not as noticeable as on MEA or MYP. From 30°C, the strain also showed rather low growth on PDA in comparison. On SNA, strain HE2 peaked at 25°C, as on MEA and MYP, but growth was rather low in comparison across all tested temperatures. The growth of strains BW1 and BW2 peaked on MEA MYP and PDA at comparatively high temperatures of 27°C or 28°C. At all measured temperatures up to 34°C, however, the growth of strain BW2 remained at a much lower level than that of BW1. Above 35°C, both strains no longer grew. Between 28°C and 31°C on MEA MYP and PDA, strain BW1 achieved the highest measured growth of all strains at this high temperatures. On SNA, both strain BW1 and BW2 culminated at 25°C to 26°C, but strain BW2 showed almost constant growth up to 29°C.

Temperature-related mortality differed only slightly between the different strains. After an incubation period of one week at 36°C and subsequent storage of the cultures at room temperature for a further two weeks, all strains grew out (**Table 3**). After the incubation period at 36°C was increased to two weeks, only twelve of the 28 cultures tested grew out after the two-week storage period. Strains NI1 and BW2 did not grow out on any culture medium during this treatment, while strains NI2 and HE2 grew on all tested culture media. None of the strains grew out after a three-week incubation period at a temperature of 36°C. Increasing the temperature to 37°C resulted in only 19 of the 28 cultures growing out after a one-week incubation period and two weeks of storage. Strains ST1 and HE2 grew out on all tested culture media during this treatment. An increase in the incubation period to two weeks and three weeks at 37°C resulted in none of the cultures showing outgrowth. After an incubation period of two weeks at a temperature of 0°C up to 2°C, none of the 28 cultures showed measurable growth (**Table 2**), however all of the cultures grew out after two weeks of storage. Increasing the temperature to 3°C resulted in 7 of the 28 cultures showing measurable growth. Strains ST1 and HE2 showed measurable growth on all culture media used except PDA. Increasing the temperature to 5°C resulted in 20 of the 28 cultures showing measurable growth. All strains showed measurable growth on all culture media used, with the exception of strains NI1 and BW2, which did not show measurable growth on any culture medium. Both strains grew out at 6 and 7 degrees, but the measured growth was at a very low level.

**Table 3:** Mortality of *Biscogniauxia nummularia* strains in culture.

	Strain	MEA	MYP	PDA	SNA		Strain	MEA	MYP	PDA	SNA
36°C	One week of incubation					37°C	One week of incubation				
	ST1	1	1	1	1		ST1	1	1	1	1
	NI1	1	1	1	1		NI1	0	1	0	0
	NI2	1	1	1	1		NI2	1	1	0	1
	HE1	1	1	1	1		HE1	1	1	1	0
	HE2	1	1	1	1		HE2	1	1	1	1
	BW1	1	1	1	1		BW1	1	1	1	0
	BW2	1	1	1	1		BW2	0	0	1	0
	Two weeks of incubation						Two weeks of incubation				
	ST1	0	1	0	0		ST1	0	0	0	0
	NI1	0	0	0	0		NI1	0	0	0	0
	NI2	1	1	1	1		NI2	0	0	0	0
	HE1	0	0	1	0		HE1	0	0	0	0
	HE2	1	1	1	1		HE2	0	0	0	0
	BW1	1	0	1	0		BW1	0	0	0	0
	BW2	0	0	0	0		BW2	0	0	0	0
	Three weeks of incubation						Three weeks of incubation				
	ST1	0	0	0	0		ST1	0	0	0	0
	NI1	0	0	0	0		NI1	0	0	0	0
	NI2	0	0	0	0		NI2	0	0	0	0
	HE1	0	0	0	0		HE1	0	0	0	0
	HE2	0	0	0	0		HE2	0	0	0	0
	BW1	0	0	0	0		BW1	0	0	0	0
	BW2	0	0	0	0		BW2	0	0	0	0

Mortality of strains (BW1-2, HE1-2, NI1-2 and ST1) is differentiated regarding to culture media (MEA, Malt Extract Agar; MYP, Malt Yeast Peptone Agar; PDA, Potato Dextrose Agar; SNA, Synthetic Nutrient-Poor Agar), temperature and incubation period. 1 = Outgrowth of the culture after incubation period and two weeks of storage at room temperature, 0 = No Outgrowth after incubation period and two weeks of storage.

### 3.2 Results of the wood decay capacities

**Figure 4:**
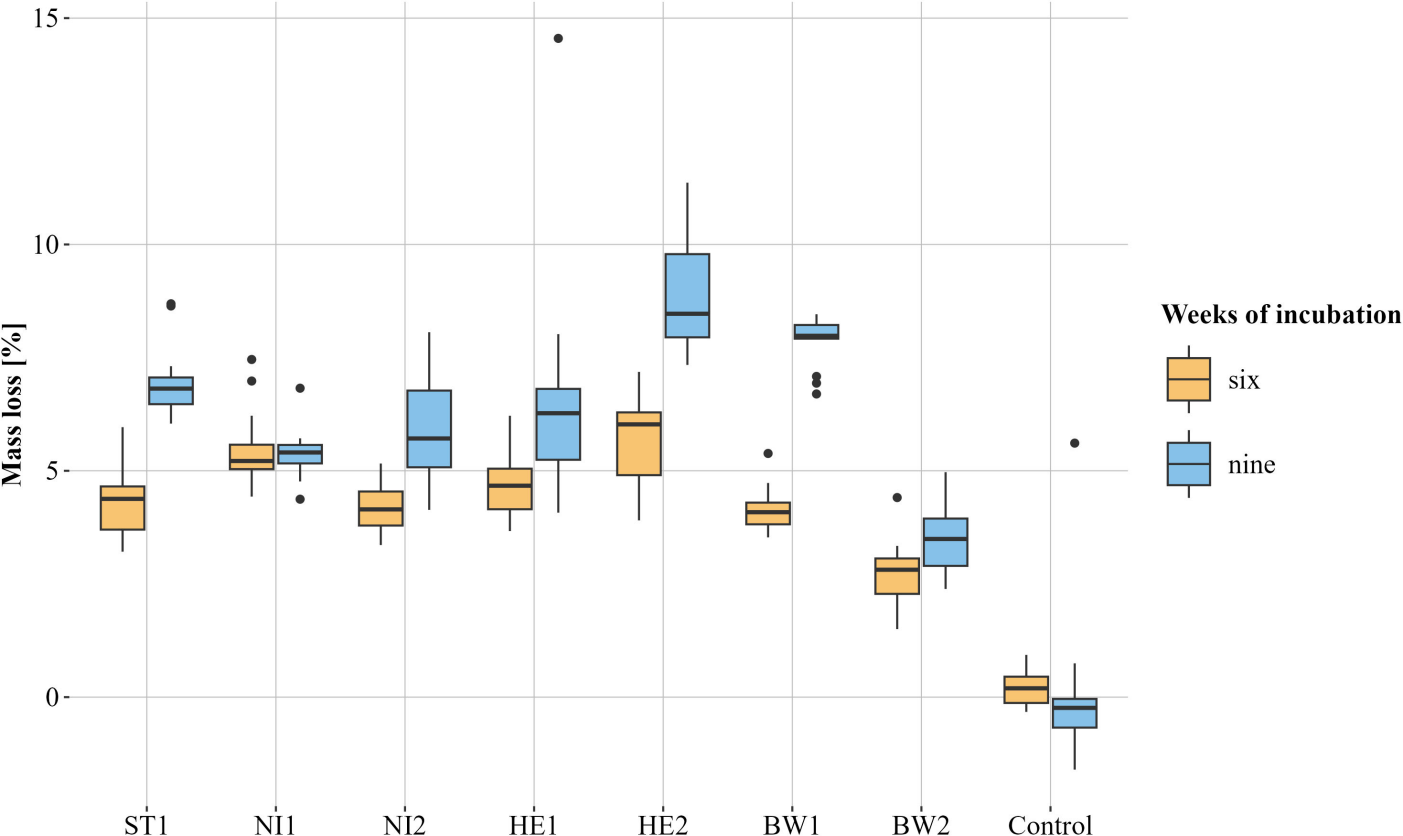
Visualisation of the relative mass loss caused by the different *Biscogniauxia nummularia* strains (BW1-2, HE1-2, NI1-2 and ST1) as box plots. The respective median is shown. Test objects that were incubated for six weeks are orange and those that were incubated for nine weeks are blue. Controls were incubated without fungus. n = 13 for each group, except for the group control, nine weeks (n = 10) and NI2, nine weeks (n = 12).

Measured temperatures ranged between 25.6°C and 25.9°C. Mass loss was observed for all TOs incubated with *B*. *nummularia* strains, with the exception of a single TO, which was incubated with strain NI2 for an incubation period of nine weeks. Therefore, the latter TO was not included in the evaluation. Additionally, three TOs from the control group with an incubation period of nine weeks could not be evaluated and were therefore also excluded. The average mass loss of the TOs across all strains was 4.5% after an incubation period of six weeks and 6.5% for the TOs that were incubated for nine weeks. An average mass loss of 0.17% was observed in the controls after both six and nine weeks. After a six-week incubation period, the mass loss of the TOs caused by the different *B*. *nummularia* strains exhibited some variation. The p-values ranged from 1 to values < 0.0001. (**Figure 4**, beta regression model and Tukey multiple comparison). On average, the greatest mass loss was observed in TOs incubated with strain HE2 (5.69%). The mass loss differed clearly – p-values between 0.0388 and < 0.0001 – from all other strains with the exception of strain NI1 (5.5%, p-value = 0.9985). Strain BW2 (2.7%) caused the lowest mass loss. It was clearly lower (p-values < 0.0001) than the mass loss caused by any other strain. In addition, the mass loss by strain NI1 was noticeably higher than that by strain ST1 (4.32%, p-value = 0.0016), NI2 (4.15%, p-value = 0.0002) and BW1 (4.16%, p-value = 0.0002). Differences in mass loss were also observed after an incubation period of nine weeks. The p-values were between 0.9801 and < 0.0001. Test objects incubated with strain HE2 showed the highest average mass loss (8.89%). The mass loss was clearly higher – p-values between 0.0031 and < 0.0001 – than the mass loss of all other strains except BW1 (7.83%, p-value = 0.4499). As with the six-week incubation period, TOs incubated with strain BW2 also showed the lowest average mass loss after nine weeks (3.56%, p-values < 0.0001). The mass loss caused by strain BW1 was considerably higher than the mass loss caused by strains NI1 (5.36%, p-value < 0.0001), NI2 (4.15%, p-value = 0.001) and HE1 (6.58%, p-value = 0.0183). Test objects incubated with strain NI1 showed a clearly lower relative mass loss than TOs incubated with strain ST1 (6.96%, p-value = 0.0069). Strain NI1 was the only strain that did not cause a higher average relative mass loss after nine weeks of incubation than after six weeks.

### 3.3 Results of the molecular analysis

Based on the ITS analysis all tested strains could be assigned to *B. nummularia*. The strains of the present study exhibit no or up to two nucleotides differences to *B. nummularia* strains MUCL 51395 (GenBank acc. NR_153649), H07 (LN714525), and BI21 (EF155488, data not shown). However, the ITS sequences of the strains analysed in this study differ in two to three nucleotides from the strain designated as *B. destructiva* (nom. inval.; GenBank acc. MT804371; Vujanovic et al., 2020).

For all strains tested, DNA sequences of the *TUB* DNA region of approximately 1800 bp were retrieved. The strains show considerable nucleotide differences between 2 and 29 nucleotides (**Supplementary Table 2**). Based on *TUB* sequence similarity four groups can be distinguished (**Figure 1**; **Supplementary Figure 1**). Group one consists of NI1, NI2, and ST1 with 3 to 6 nucleotide differences between them. Group two consists of BW1 and HE1 with 2 nucleotide differences between them. Both BW2 and HE2 have at least 17 nucleotide variations from every other strain that was examined.

## 4 Discussion

The results of the present study show that the temperature-related growth of *B*. *nummularia* can differ considerably between strains *in vitro*. However, it must be taken into account that the density of the colony cannot be inferred from the diameter growth of the colony (Wells and Uota, 1970). The growth extent of some of the tested strains was observed to reach its maximum at 25°C on the tested culture media. In contrast, the optimum growth temperature for other strains was found to be 28°C. Since temperatures between 8 and 24°C were not tested in the present study, it is possible that a few of the strains tested would have reached their maximum measured growth on certain media at even lower temperatures. Overall, the growth of the strains for temperatures between 25 and 32 degrees differed considerably less on SNA than on the other culture media tested. This is probably due to the fact that SNA is a nutrient-deficient medium compared to the other culture media used. There were also differences between the strains with regard to the lowest and highest temperature at which growth was measurable. Depending on culture medium, some strains appear to have a considerably wider temperature range in which measurable growth is possible than others. Strain ST1, for example, showed measurable growth on MEA at 3°C up to 34°C, while strain NI1 only showed measurable growth between 6°C and 31°C. Strains that did not show measurable growth at temperatures under 5°C or 6°C tended to reach their maximum growth at temperatures above 26°C, at least on most of the tested culture media. Examples of this are strains BW1 and BW2 from Baden-Württemberg and, to a lesser extent, strain HE1 from southern Hesse. For all localities from which strains were isolated the average summer temperature (June, July, and August) for the reference period 1991 to 2020 was compared (**Supplementary Table 2**). According to this comparison the latter strains originated from localities with higher average summer temperatures. The values were 19.2°C for strain BW1 and 18.6°C for strain BW2. At 17.8°C, the value of the closest weather station for strain HE1 is at least slightly higher than the rest of the strains (17.0°C to 17.6°C). So it is possible that there is an influence of the site and that *B*. *nummularia* strains that have adapted to the higher temperatures are becoming established in southern Germany. Patejuk et al. (2022) report indications for a northern population of the fungus, which might be adapted better to the Central European climate. In contrast to the strains BW1 and BW2 strain NI1 (Lower Saxony) did not show measurable growth for temperatures under 6°C and yet achieved its greatest growth already at 25°C at least on MEA. Overall, strain NI1 appears to be a strain with a very small temperature-related growth amplitude.

There were slight differences at temperatures (above the optimal temperature), which led to the death of the various strains. After an incubation period of one week at 36°C, all strains still grew out. Increasing the temperature to 37°C resulted in some strains no longer surviving on all tested media after a one-week incubation period. Here too, differences between strain ST1 and strain NI1 became apparent. Strain NI1 died on all tested media except MYP, while strain ST1 did not die on any medium. An increase in the incubation period to two weeks at 36°C resulted in strain ST1 dying on all media except MYP. Strains NI2 and HE2 survived under these conditions. Strain HE2 in particular reached its maximum growth on most media at comparatively low temperatures.

Differences were observed between the wood decay capacities of the various strains tested. After both, six and nine weeks of incubation, the TOs incubated with strain HE2 exhibited the greatest average relative mass loss. For both incubation periods, this relative mass loss differed clearly from most of the other strains tested. TOs incubated with strain BW2 exhibited the lowest average relative mass loss after both, six and nine weeks of incubation. This mass loss was clearly different from all other strains for both incubation periods. The reasons why the strains differ so much in their wood decay capacities can only be surmised at this point. Numerous authors have shown that strains of one fungal species can differ in their pathogenicity towards their host (*e.g.* Elgersma and Heybroek, 1979; Jarosz and Davelos, 1995; Lee et al., 2015; Ghelardini et al., 2016). It is possible that the HE2 strain is more virulent than the BW2 strain. However, as the study was carried out *in vitro*, the results cannot simply be used to draw conclusions about the pathogenicity of the fungal strains in living tissue. Host-pathogen relationships are highly complex. Due to a variety of influencing factors, it is impossible to predict the exact growth rate of a fungus in a living tree (Schwarze et al., 1999). It is noteworthy that the strain HE2, which caused the highest degree of wood degradation, also showed the greatest growth on almost all culture media at 25°C. The optimum growth temperature for strain BW2 was 28°C on almost all media. It is possible that if the incubation temperature had been set at 28°C, strain BW2 would have caused a greater relative mass loss. Strain BW1, which had similarly high optimum growth temperatures as strain BW2 depending on the medium, also caused a comparatively low relative mean mass loss after six weeks. However, the average relative mass loss after nine weeks was almost twice as high. This contrasts with strain NI1, for which the relative mass loss caused did not increase on average between six and nine weeks of incubation. In conjunction with the results of the growth experiment, it can be assumed that the wood decay capacity of each strain is temperature-dependent since the optimal temperature for hyphal growth varies between strains. Nevertheless, this is merely a single factor, and it can be assumed that a multitude of factors contribute to the wood decay capacities of *B*. *nummularia*. However, the data presented in this study demonstrate that the host species from which the strain was isolated has no obvious impact on its capacity to decay European beech wood. Strain NI2 (*F. sylvatica*) does not differ noticeably from strains ST1 (*P. sylvestris*) and HE1 (*P. menziesii*) after either six or nine weeks of incubation. Both, the strain causing the highest relative mass loss (HE2) and the strain causing the lowest relative mass loss (BW2) were isolated from European beech.

To our surprise, notable differences were found in the tubulin DNA region of the tested strains, with high similarities between strains isolated from plots located within distinct circular radii, implying a geographic component. These groups are not necessarily reflected in the results of the growth or wood decay capacities. The findings are at odds with those of Patejuk et al. (2022), who discovered no noteworthy variations in the beta tubulin region with a considerably larger number of strains but a much smaller number of compared base pairs (using primer pair Bt2a + Bt2b, representing a approx. 450 bp region). The tubulin region is usually one of the more conserved gene coding regions (Sullivan and Cleveland, 1986). The observed dissimilarities can be primarily explained by differences in intron regions. Additionally, for a number of fungal species, the presence of paralogue or pseudogene regions is known in association with the tubulin region (May et al., 1987; Ayliffe et al., 2001; Hubka and Kolarik, 2012). Unfortunately, since there is currently little data available on the tubulin DNA region of *B. nummularia* from other strains and localities, and only seven strains were tested in the study, this apparent phenomenon at present cannot be investigated in more detail. However, the observations presented in this study justify reassessing the species concept of *B. nummularia* while considering intraspecific variability. Ideally, examination of the phylogenetic relationships between different populations should be done over a larger geographic region, using additional markers and an adequate number of isolates.

In conclusion, the present study is the first to show that *Biscogniauxia nummularia* strains can differ in their temperature-related growth characteristics. There are indications that the temperature-related differences could be related to the origin of the respective strain and therefore the site. For future studies, to gain a more comprehensive understanding of the subject matter, it would be beneficial to analyse a greater number of strains across an even larger geographical area. Furthermore, including multiple strains isolated from the same site would facilitate a more in-depth investigation into the influence of the site. The results of the present study demonstrate the variability in the temperature-related growth characteristics and the wood decay capacities of *B*. *nummularia* strains, thereby contributing to a more comprehensive understanding of VLB.

## Data Availability

The datasets presented in this study can be found in online repositories. The names of the repository/repositories and accession number(s) can be found in the article/**Supplementary Material**.

## Footnotes

1
indexfungorum.org (retrieved 03.12.2024).

2
https://meteostat.net/de/ (retrieved 30.11.2024).

## Supplementary material

Supplementary Figure 1 Unrooted tree retrieved from Maximum likelihood phylogenetic analysis of *Biscogniauxia nummularia* TUB sequence alignment. ML bootstrap support values above 60% are shown at the branches.
