# Supplementary figures and images for: Temperature-related growth limits and wood decay capacity of the warmth-loving fungus Biscogniauxia nummularia in vitro
Source: Front Fungal Biol. 2025 Apr 11;6:1548128. doi: 10.3389/ffunb.2025.1548128 (PMC12021901; doi:10.3389/ffunb.2025.1548128)

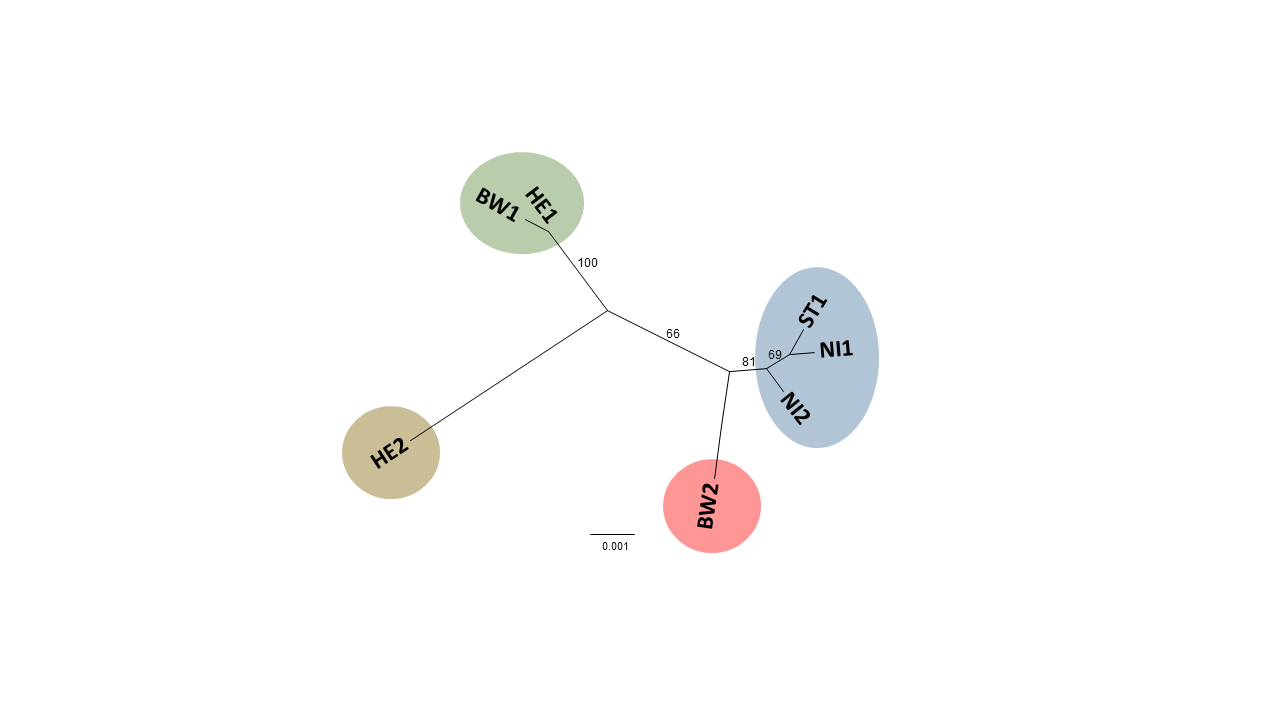

Supplement: Supplementary Figure 1 — Unrooted tree retrieved from Maximum likelihood phylogenetic analysis of Biscogniauxia nummularia TUB sequence alignment. ML bootstrap support values above 60% are shown at the branches. [file Image1.tif]
